# Outlier Detection in Urban Air Quality Sensor Networks

**DOI:** 10.1007/s11270-018-3756-7

**Published:** 2018-03-08

**Authors:** V. M. van Zoest, A. Stein, G. Hoek

**Affiliations:** 1 Faculty of Geo-Information Science and Earth Observation (ITC), University of Twente, PO Box 217, 7500 AE Enschede, The Netherlands; 2 Institute for Risk Assessment Sciences (IRAS), Utrecht University, PO Box 80178, 3508 TD Utrecht, The Netherlands

**Keywords:** Air quality, Air pollution, Outlier detection, NO_2_, Sensor network

## Abstract

Low-cost urban air quality sensor networks are increasingly used to study the spatio-temporal variability in air pollutant concentrations. Recently installed low-cost urban sensors, however, are more prone to result in erroneous data than conventional monitors, e.g., leading to outliers. Commonly applied outlier detection methods are unsuitable for air pollutant measurements that have large spatial and temporal variations as occur in urban areas. We present a novel outlier detection method based upon a spatio-temporal classification, focusing on hourly NO_2_ concentrations. We divide a full year’s observations into 16 spatio-temporal classes, reflecting urban background vs. urban traffic stations, weekdays vs. weekends, and four periods per day. For each spatio-temporal class, we detect outliers using the mean and standard deviation of the normal distribution underlying the truncated normal distribution of the NO_2_ observations. Applying this method to a low-cost air quality sensor network in the city of Eindhoven, the Netherlands, we found 0.1–0.5% of outliers. Outliers could reflect measurement errors or unusual high air pollution events. Additional evaluation using expert knowledge is needed to decide on treatment of the identified outliers. We conclude that our method is able to detect outliers while maintaining the spatio-temporal variability of air pollutant concentrations in urban areas.

**Electronic supplementary material:**

The online version of this article (10.1007/s11270-018-3756-7) contains supplementary material, which is available to authorized users.

## Introduction

Air quality is monitored globally, with national monitoring networks being used to assess air pollution in relation to environmental limit values. In Europe, national, regional, and local environmental agencies operate these monitoring networks according to EU guidelines (European Parliament and Council of the European Union 2008), complying to high standards of equivalency (EC Working Group on GDE 2010). Each European country has a network of air quality monitoring stations that are located in urban, suburban, and rural areas.

Health effects of air pollution have attracted public and scientific attention globally as the global burden of disease of outdoor air pollution is significant (Cohen et al. 2017). The health risks are typically highest in urban areas because of their high population density, a high density of schools and hospitals, and higher air pollution concentrations. In recent local networks, urban air quality is measured using a larger number of sensors than in national air quality networks, allowing detection of more local sources. In response to the increasing civil interest in the air they breathe, more local initiatives have resulted in extended low-cost monitoring networks. These provide more detailed spatio-temporal data on air quality. Data from such sensor networks however are more prone to result in errors, and their spatio-temporal data quality is often unknown (Snyder et al. 2013). This leads to an increased need for data evaluation. Data evaluation of low-cost air quality networks typically includes outlier detection, comparison with classical monitors, comparison of inter-sensor measurements, and evaluation of the stability of sensors. In this paper, we focus on outlier detection.

Outlier detection is an important part of data cleaning and particularly relevant for low-cost air quality sensor networks. Outlier detection is defined as the detection of values that are statistically significantly different from the expected value at a given time and location. Outlier detection is important not only for detecting air pollution events but also for removing errors that might otherwise affect data analysis and comparison, including unnecessary unrest among the population if data are publicly available online. Errors in this context refer to inaccuracies due to air quality sensor faults, mistakes in the human handling of the sensors, or positioning of the sensors under conditions for which they are not designed. Events are valid observations of very high or low air pollutant concentrations compared to the concentrations expected at a given time in a given location (Zhang et al. 2007). True events can be related to very local sources (e.g., a small fire, truck idling within meters of a monitor) or to very unusual weather circumstances such as low mixing height and high atmospheric stability resulting in poor dispersion of emitted pollutants.

Functional outlier detection, as a common type of temporal outlier detection, compares various function curves of fixed time periods. In the past, this method was applied to PM_10_, SO_2_, NO, NO_2_, CO, and O_3_ to detect months with unusually high air pollutant concentrations (Martínez Torres et al. 2011), or to detect working days and non-working days with outlying NO_x_ levels (Febrero et al. 2007, 2008; Sguera et al. 2016). Functional outlier detection is used to compare entire vectors of measurements (e.g., all observations in a month) and is therefore less suitable for the detection of individual outliers. Comparing an observation only to its temporal neighborhood may also lead to the neglect of a systematic bias in the sensor.

In spatial outlier detection, an observation is compared to the observations in its spatial neighborhood. Bobbia et al. (2015) used kriging to detect outliers in PM_10_ concentrations on a provincial scale. Spatio-temporal outlier detection combines the spatial neighborhood with a temporal neighborhood. It has been applied to PM_10_ measurements at the European scale (Kracht et al. 2014). At this scale level, however, only rural and urban background stations can be used, as the methods are not suitable for dealing with the wide spatial variation of air pollutants in an urban area.

For an urban air quality sensor network, both spatial and spatio-temporal outlier detection have only been applied to air pollutants that show a low spatial variation. Hamm (2016) and Shamsipour et al. (2014) applied spatial and spatio-temporal outlier detection methods on PM_10_, which in cities is mostly dominated by regional background concentrations from sources outside the city (Eeftens 2012). Distance-weighting techniques such as kriging were successfully applied to urban PM_10_ for filling missing values and for outlier detection. There was no need for space varying covariates because PM_10_ concentration was not related to the type of location or street (Hamm 2016). For NO_2_, however, the concentrations can vary over short distances, e.g., governed by the traffic density of a street (Briggs 1997; Cyrys 2012). As the distances over which NO_2_ concentrations vary (tens of meters) are commonly shorter than the distances between sensor locations (kilometers), spatial outlier detection methods based on distance-weighting cannot be applied to NO_2_ measurements in cities.

The objective of this study was to develop an adequate outlier detection method for an urban air quality sensor network. Such a network is characterized by a fine-scale spatial and temporal variation in air quality. For this study, we use NO_2_ data from an air quality sensor network located in the city of Eindhoven, the Netherlands.

## Data Preprocessing

**Fig. 1:**
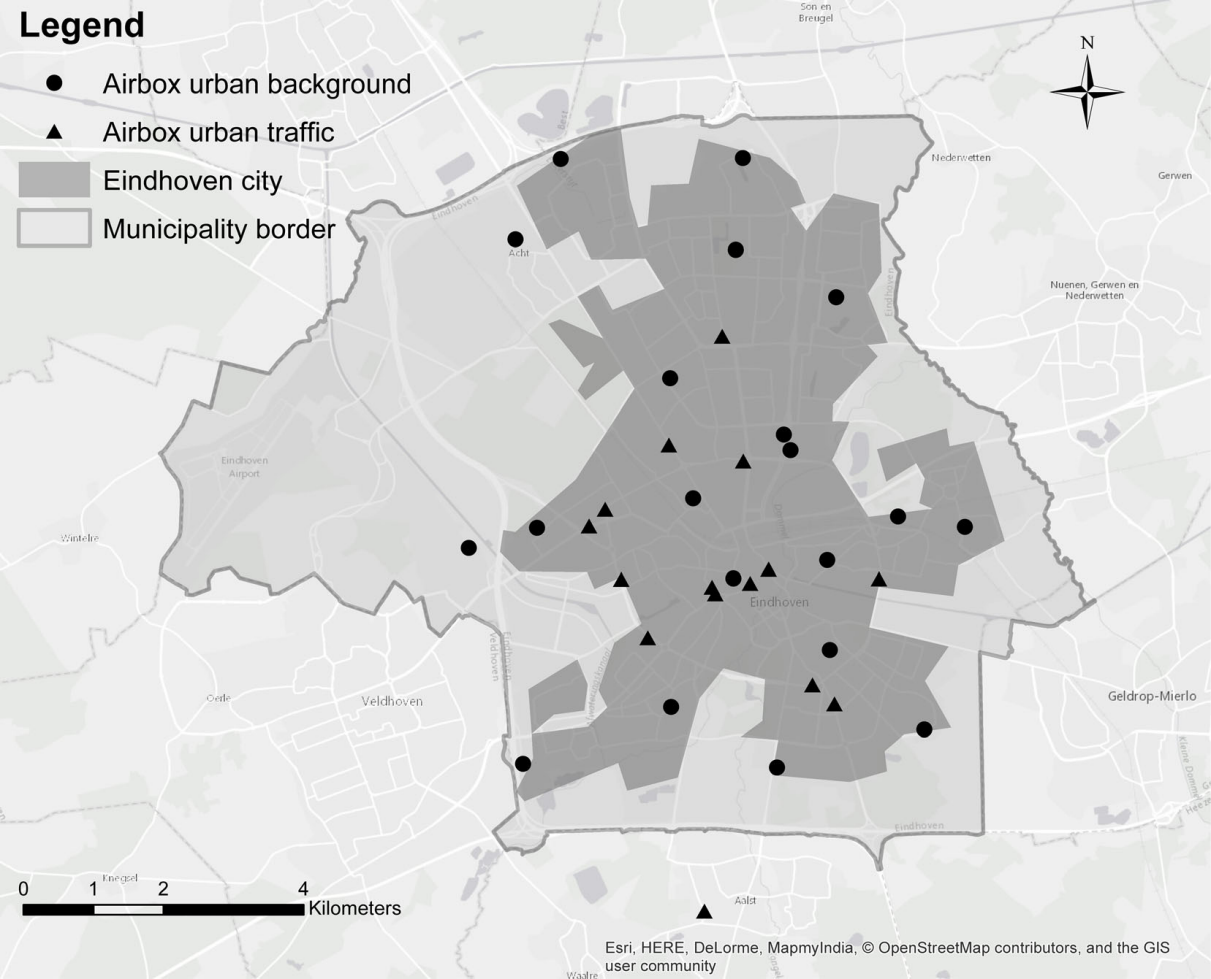
Locations of the airboxes in the city of Eindhoven, the Netherlands, at urban background locations (circles) and urban traffic locations (triangles)

The air quality sensor network in Eindhoven (Fig. 1) was established by the AiREAS civil initiative (Close 2016), and is the first fine resolution urban air quality sensor network in the Netherlands. It was installed in November 2013 and has been operated continuously since. The network consists of 35 weatherproof airboxes of size 43 × 33 × 20 cm, containing an array of sensors. Each airbox measures particulate matter, ozone (O_3_), and/or nitrogen dioxide (NO_2_) and also temperature and humidity as the air flows through (Hamm et al. 2016). The airboxes have a fixed position and are attached to lamp posts for power supply.

We focus on NO_2_, as an air pollutant with a high spatial variability in urban areas (Cyrys 2012). The hourly concentrations measured by the conventional monitors in Eindhoven ranged from 2.5 to 123.8 μg m^−3^ in 2016, with a mean of 28.6 μg m^−3^ and a standard deviation of 16.5 μg m^−3^. The distribution of NO_2_ concentrations is skewed with a long right tail (P_95_ = 61.0 μg m^−3^, P_99_ = 78.8 μg m^−3^). The airboxes measure NO_2_ concentrations using a Citytech Sensoric NO_2_ 3E50 sensor adapted by the Energy Research Center of the Netherlands (ECN). The concentration of air pollutants is measured every 10 min. The data are sent to a server using a GPRS connection (Hamm et al. 2016). To reduce the noise, the 10-min NO_2_ measurements were averaged to hourly values for the current analysis. Data for the full year of 2016 were used for this study. The sensors were calibrated at the end of 2015.

The data were cleansed before being used. Negative concentration values occurred when the concentrations were below the limit of detection and were removed from the dataset (1.5%). Zeroes in the data indicated a sensor failure and were removed from the dataset (1%). High peaks in NO_2_ concentrations can occur in 10-min data if the sensor is exposed to a high concentration peak for a short period of time. Similar peaks in hourly concentration data however are more likely to be caused by sensor failure and influence the outlier detection. To carefully remove extreme peaks in hourly concentrations, we turned to the two conventional NO_2_ monitors in Eindhoven, which are part of the national air quality monitoring network. We set a threshold equal to three times the maximum hourly concentration measured in 2016. In doing so, concentration values *x*_*i*_ > 372 μg m^−3^ were removed (0.02%). Such extreme peaks are impossible to occur under natural conditions in this city and are most probably caused by sensor failures. Such failures also caused frozen concentration values for several hours or days. Those values were removed from the dataset as well (1.5%). One airbox showed a consistent positive bias. Including it in the analysis not only showed the many outliers of the airbox but also strongly influenced the percentage of outliers that could be detected in other airboxes, which almost dropped to zero. Therefore, data of this airbox was removed prior to the final outlier detection shown here.

## Methods

Outlier detection is based upon checking whether an observed concentration value falls within a given confidence interval, set by

1 $$ \mu \pm z\times \sigma $$

where *μ* is the mean NO_2_ concentration level in μg m^−3^, *σ* is the standard deviation, and *z* is an indicator of the size of the confidence interval. We consider Eq. (1) for grouped NO_2_ concentration observations within temporal, spatial, and spatio-temporal neighborhoods. Assuming independence and normality, then the value of *z* is set at 1.96 for a 95% confidence level (Kracht et al. 2014) or at 2.97 for a 99.7% confidence interval, depending on the required strictness of the outlier detection. We used *z* = 2.97, which in related studies has been rounded to *z* = 3 (Martínez Torres et al. 2011; Shamsipour et al. 2014).

NO_2_ concentrations in an urban setting, however, highly depend on the proximity of busy roads, and therefore, too much noise in concentrations is found within the neighborhood to detect values that are abnormally high given their location. Similarly, temporal neighborhoods have a highly temporally dependent variation in air pollutant concentrations over the day.

We propose to overcome this by classifying the locations and time periods into 16 spatio-temporal categories distinguished by different levels of air pollution. To do so, we divided the measurement locations into two categories: urban traffic and urban background locations. These take into account the positions of the airboxes near specific land use types, the presence of traffic, and distance from the center. We take four intervals: traffic hours (6:01–9:00 and 16:01–20:00 UTC time), off-peak hours (9:01–16:00 and 20:01–22:00 UTC time), transition periods (22:01–1:00 and 5:01–6:00 UTC time), and night hours (1:01–5:00 UTC time).

Days of the week were divided into two classes: weekdays (Monday to Friday) and weekend days (Saturday and Sunday). This all resulted into 16 classes: eight temporal classes and two spatial classes. For each spatio-temporal class *K*, the three steps described below are taken to detect outliers.

**Fig. 2:**
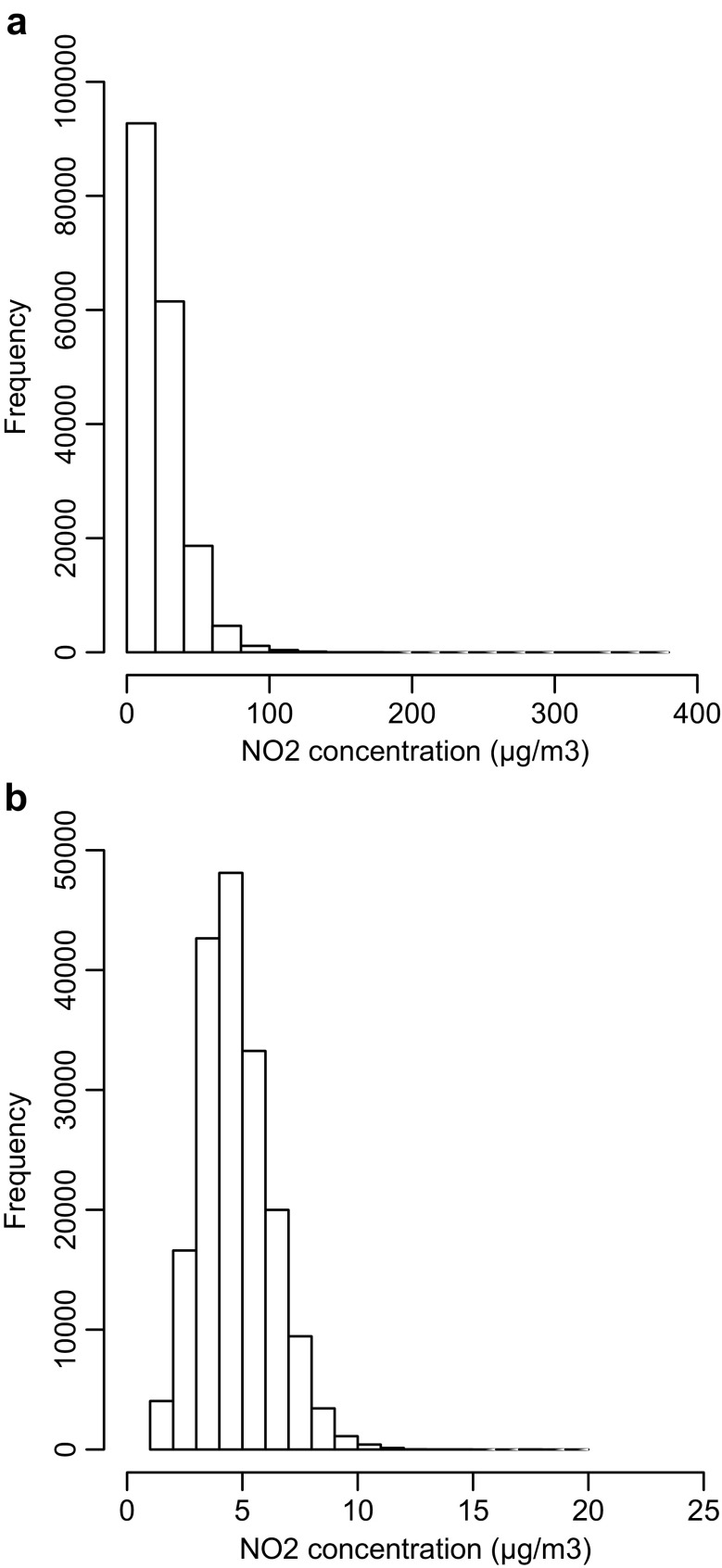
Distribution of NO_2_ concentrations **a** before square root transformation and **b** after square root transformation

1. We transformed the NO_2_ concentrations using the square root transformation to obtain approximately normally distributed values (Fig. 2), i.e., to justify the use of Eq. (1).

Before transforming the NO_2_ concentration values, in line with Kracht et al. (2013), we added a value of (1 − minimum value of all observations) to all observations to prevent values < 1 μg m^−3^ from increasing during square root transformation while values > 1 μg m^−3^ decrease:

2 $$ {x}_c=\sqrt{NO{2}_c+\left(1-\min \left( NO{2}_c\right)\right)} $$

where NO2_*c*_ is an observation and *x*_*c*_ is the transformed observation in spatio-temporal class *K*, where $K={\bigcup}_{c\in C}\left({x}_c\right)$ and *c* is an observation index in *C* = {1…*N*_*C*_} for *N*_*C*_ total number of observations in class *K*. Note that *x*_*c*_ has coordinates in space and time.

**Fig. 3:**
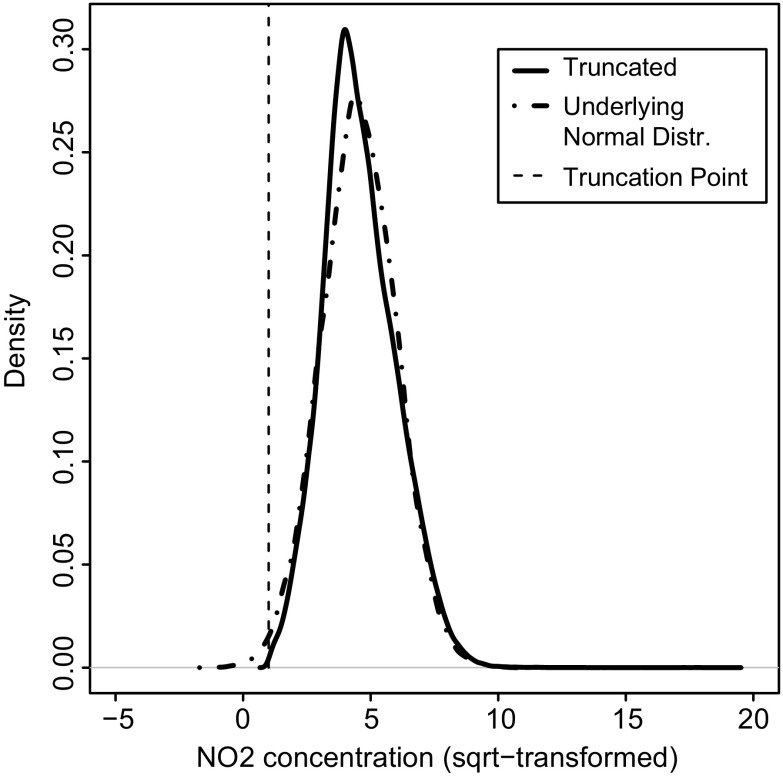
The truncated normal distribution of square-root-transformed NO_2_ concentrations (solid line) and its underlying normal distribution (dot dashed line). The truncation point is set at 1 (dotted line)

2. As a result of the transformation in Eq. (2), the distribution of NO_2_ concentrations is truncated at the left at 1 μg m^−3^. The resulting distribution thus showed a truncated normal distribution (Fig. 3).

For each square-root-transformed NO_2_ observation *x*_*c*, *i*_, we temporarily excluded the *i*th observation from the NO_2_ concentration dataset in order to avoid impact of the observation, a potential outlier, on the standard deviation and mean. We then obtained the mean and standard deviation of the remainder of the dataset as

3 $$ {m}_K^{-i}=\frac{\sum_c\left({x}_c\right)-{x}_{c,i}}{\left({N}_C-1\right)} $$

4 $$ {s}_K^{-i}=\sqrt{\frac{\sum_c{\left({x}_c-{m}_K^{-i}\right)}^2-{\left({x}_{c,i}-{m}_K^{-i}\right)}^2}{\left({N}_C-2\right)}} $$

where summation extends over all hourly NO_2_ observations *x*_*c*_ in one spatio-temporal class *K* and ${m}_K^{-i}$ and ${s}_K^{-i}$ are the mean and the standard deviation of all hourly NO_2_ observations excluding the *i*th observation *x*_*c*, *i*_, respectively. Note that *c*, *i* ∈ *C* and *N*_*C*_ is the total number of observations in class *K*.

Equations (3) and (4) provided both the mean and the standard deviation of the truncated normal distribution of NO_2_ concentrations, referred to as ${m}_K^{-i}$ and ${s}_K^{-i}$. Equation (1) requires a normal distribution, and therefore, we are more interested in the mean and standard deviation of the underlying normal distribution, referred to ${n}_K^{-i}$ and ${t}_K^{-i}$, respectively, rather than the mean and standard deviation of the truncated normal distribution. We use a maximum likelihood estimator to obtain estimated values ${n}_K^{-i}$ and ${t}_K^{-i}$. The log likelihood function is given as

5 $$ {\sum}_c\ln \left(f\left({x}_c|\theta \right)\right) $$

where *f*(*x*_*c*_| *θ*) is the probability density function of the truncated normal distribution of NO_2_ concentrations, returning the probability of observing *x*_*c*_ given a set of parameters $\theta =\left({m}_K^{-i},{s}_K^{-i},a,b\right)$, for *a* ≤ *x* ≤ *b*. In our case of left truncation, we have *a* = 1 and *b* = ∞. Then, the probability density function is given as

6 $$ f\left({x}_c|\theta \right)=\frac{\phi \left(\frac{x_c-{n}_K^{-i}}{t_K^{-i}}\right)}{t_K^{-i}\left(1-\Phi \left(\frac{a-{n}_K^{-i}}{t_K^{-i}}\right)\right)} $$

Imputing Eq. (6) into the log likelihood function and taking${\theta}_1=\left({n}_K^{-i},{t}_K^{-i}\right)$ gives

7 $$ L\left({\theta}_1\right)={\sum}_c\left(\ln \left(\phi \left(\frac{x_c-{n}_K^{-i}}{t_K^{-i}}\right)\right)-\ln \left({t}_K^{-i}\left(1-\Phi \left(\frac{a-{n}_K^{-i}}{t_K^{-i}}\right)\right)\right)\right) $$

where *ϕ*(∙) is the probability density function of the normal distribution and *Φ*(∙) is the corresponding cumulative distribution function. Optimization of the log likelihood function Eq. (7) using Nelder and Mead (1965) gives maximum likelihood values for ${n}_K^{-i}$ and ${t}_K^{-i}$. We used the parameters ${m}_K^{-i}$ and ${s}_K^{-i}$ as starting values.

For each observation *x*_*c*, *i*_ removed from the dataset, ${n}_K^{-i}$ and ${t}_K^{-i}$ are computed on the remainder of the spatio-temporal class dataset as described above.

3. Next, Eq. (1) is adapted to find the lower and upper thresholds of values considered outliers:

8 $$ {n}_K^{-i}\pm z\times {t}_K^{-i} $$

which is computed for each individual observation. If the *i*th observation *x*_*c*, *i*_ falls outside this interval, it is considered to be an outlier. The observations of spatio-temporal class *K* are backtransformed after the outlier detection:

9 $$ NO{2}_c={\left({x}_c\right)}^2-\left(1-\min \left({x}_c\right)\right) $$

returning the NO_2_ concentrations in μg m^−3^. Depending upon the purpose of the outlier detection, the outlying observations can then be removed or further investigated.

We further computed the thresholds for the entire dataset, without removal of observation *x*_*c*, *i*_ in Eqs. (3) and (4). The mean and standard deviation of the underlying normal distribution are then expressed by *n*_*K*_ and *t*_*K*_, respectively, which results in the following thresholds:

10 $$ {n}_K\pm z\times {t}_K $$

which are also back-transformed using Eq. (9). These thresholds are not used for actual outlier detection, but as an approximation of the thresholds for each spatio-temporal class. This allowed us to compare the thresholds of the 16 spatio-temporal classes. Given the large number of observations in each class, the thresholds are not highly affected by removing one of the observations.

For comparison with conventional monitors, the same analysis was repeated with data from the two NO_2_ monitors in Eindhoven which are part of the national air quality monitoring network. Both conventional monitors are located in an urban traffic location and therefore considered as the same spatial class. We used the temporal classification similar to the one used in the analysis of the airbox data.

## Results

Of the 25 airboxes measuring NO_2_ that were used for this analysis, 11 were classified as urban background locations, and 14 were classified as urban traffic locations. Table 1 shows the approximated upper thresholds for outliers in each spatio-temporal class (Eq. (10)). All lower thresholds were equal to zero. For the values of *n*_*c*_ and *t*_*c*_ of each spatio-temporal class, we refer to Table S1 in the supplementary materials. Table 2 shows the percentage of outliers detected per spatio-temporal NO_2_ concentration class using a full year of hourly NO_2_ data. Note that our method defines unusual observations, which are not necessarily errors, but which could also be very unusual air pollution events related to local sources, or extreme weather conditions of low wind speed and high atmospheric stability.

**Table 1:** Upper thresholds for hourly average NO_2_ concentrations (μg m^−3^) above which considered outliers, per spatio-temporal class, using *z* = 2.97

	Urban traffic		Urban background	
	Week	Weekend	Week	Weekend
Rush hours	96.6 (*n* = 17,761)	78.4 (*n* = 7,127)	81.0 (*n* = 17,660)	62.3 (*n* = 6,983)
Off-peak hours	87.3 (*n* = 22,768)	76.7 (*n* = 9,153)	72.9 (*n* = 22,554)	61.3 (*n* = 8,961)
Night hours	63.2 (*n* = 10,161)	63.6 (*n* = 4,123)	58.6 (*n* = 9,983)	57.3 (*n* = 3,995)
Transition hours	76.5 (*n* = 10,195)	67.1 (*n* = 4,129)	67.9 (*n* = 10,031)	56.4 (*n* = 3,983)

Between brackets, n shows the number of hourly concentration values in this class

**Table 2:** Percentage outliers per spatio-temporal NO_2_ concentration class for hourly values in 2016, using *z* = 2.97

	Urban traffic		Urban background	
	Week	Weekend	Week	Weekend
Rush hours	0.2%	0.2%	0.2%	0.2%
Off-peak hours	0.2%	0.2%	0.2%	0.2%
Night hours	0.2%	0.5%	0.1%	0.5%
Transition hours	0.3%	0.3%	0.3%	0.3%

Table 2 shows that the period of night hours during the weekend has an increase in the number of outliers, both for urban traffic locations and urban background locations. Both *n*_*c*_ and *t*_*c*_ are relatively small in these spatio-temporal classes compared to other spatio-temporal classes. The combination of a short right tail and the relatively small *n*_*c*_ and *t*_*c*_ cause the upper threshold to be low while detecting a relatively high number of outliers in the thicker tail. All categories have an approximately similar percentage of outliers and there are no large deviations.

**Fig. 4:**
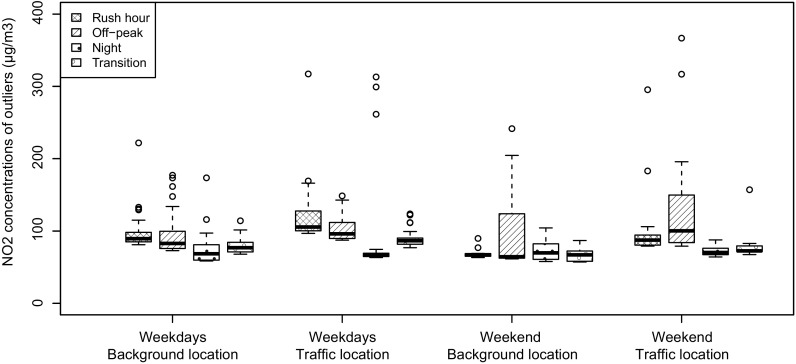
Boxplots of the outliers in each spatio-temporal class

The boxplots in Fig. 4 show the range in concentrations that were considered outliers for each spatio-temporal class. The lower whiskers are short and close to the threshold values shown in Table 1. Especially during off-peak hours in the weekend, the range in concentrations of the outliers is large. Extreme outliers, denoted by the dots, representing observations outside 1.5 × IQR (interquartile range) of the outliers, occur in many spatio-temporal classes. Note that these boxplots are only based on the outliers, which is a small number of observations.

**Fig. 5:**
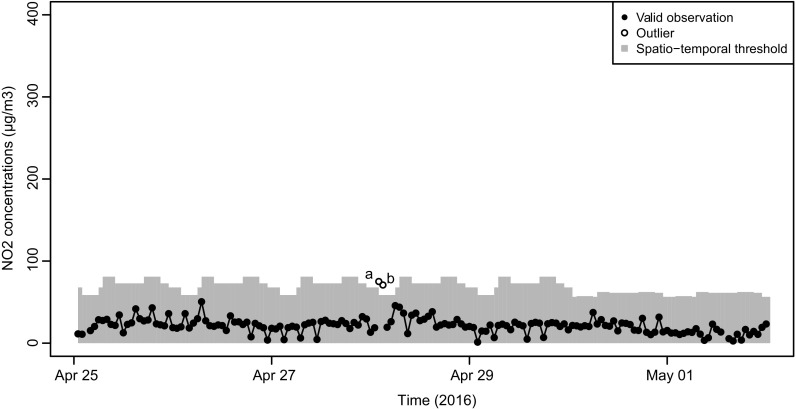
NO_2_ concentrations measured by airbox 6, an urban background location. Filled circles indicate non-outlying observations; unfilled circles indicate outliers using *z* = 2.97. The gray bars indicate the threshold values for each temporal class, for urban background airboxes

**Fig. 6:**
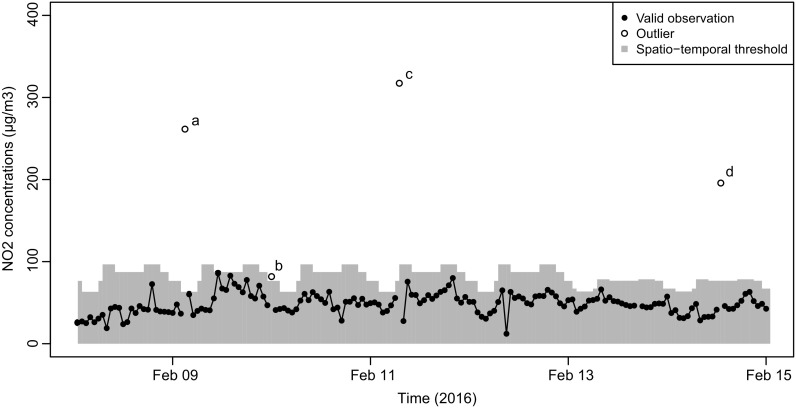
NO_2_ concentrations measured by airbox 26, an urban traffic location. Filled circles indicate non-outlying observations; unfilled circles indicate outliers using *z* = 2.97. The gray bars indicate the threshold values for each temporal class, for urban traffic airboxes

Figures 5 and 6 show NO_2_ measurements during 2 weeks in 2016 containing outliers. Figure 5 shows the week from April 25 until May 1, of an urban background location, whereas Fig. 6 shows the week from February 8 until February 14 of an urban traffic location. The concentrations at the urban traffic location were higher than those at the urban background location. Due to the spatial classification, some concentration values are considered outliers at the urban background location, while they are non-outliers at the urban traffic location. The temporal classification is also visible in Fig. 6: concentration values that are considered outliers at one point in time can be considered non-outliers at other points in time, e.g., during rush hours in which higher concentrations are expected. This is a major difference as compared to applying the outlier threshold on the entire dataset without classification (Eq. (1)), yielding an expected 0.3% of outliers as cutoff peaks without taking spatio-temporal variability in the NO_2_ concentrations into account.

Figure 5 shows two outliers, labeled (a) and (b), occurring during the night, in the early morning (1:00–3:00) of April 28. During weekday night hours at an urban background location, the transformed (Eq. (2)) parameter estimations are *n*_*c*_ = 3.965 and *t*_*c*_ = 1.265. Entered in Eq. (8) with *z* = 2.97, and back transformed using Eq. (9), this gives an upper threshold of 58.6 μg m^−3^. The concentrations measured at outliers (a) and (b) were 75 and 70.8 μg m^−3^, respectively, both exceeding the upper threshold. Given that these are consecutive observations and within the range of thresholds of other periods, it is not clear whether these observations reflect instrument error.

From Fig. 6, we identify four outliers, labeled (a)–(d). Three outliers, specifically (a), (c), and (d), are clearly higher than expected concentration values in any of the spatio-temporal categories. They are furthermore single observations. Outlier (b) occurred on February 9 from 23:00 to 0:00 in the temporal class “transition period.” In this spatio-temporal class, with (transformed) *n*_*c*_ = 4.76 and *t*_*c*_ = 1.36, the upper threshold is approximately (4.76 + 2.97 × 1.36)^2^ − (1 − 0.0244) = 76.5 μg m^3^. The concentration measured at (b) is 81.8 μg m^−3^, exceeding the upper threshold. However, during the daytime, such a concentration value would have been within expected concentration values.

There was seasonal deviation in the number of outliers: a higher number of outliers was detected in spring (0.37%) compared to the mean percentage of outliers of the entire year (0.22%). In summer, the number of outliers was relatively low (0.09%).

Table 2 shows no difference in the percentage of outliers between urban traffic locations and urban background locations. Some individual airboxes however show more outliers than others. Most airboxes have 0–0.1% outliers for a year of data, whereas a few airboxes have a larger percentage of outliers for some spatio-temporal classes, up to a maximum of 2.5% for one airbox for one spatio-temporal class. The highest percentages of outliers are found in airboxes with the highest mean concentration values. The percentage of outliers of an airbox varies between spatio-temporal classes.

**Fig. 7:**
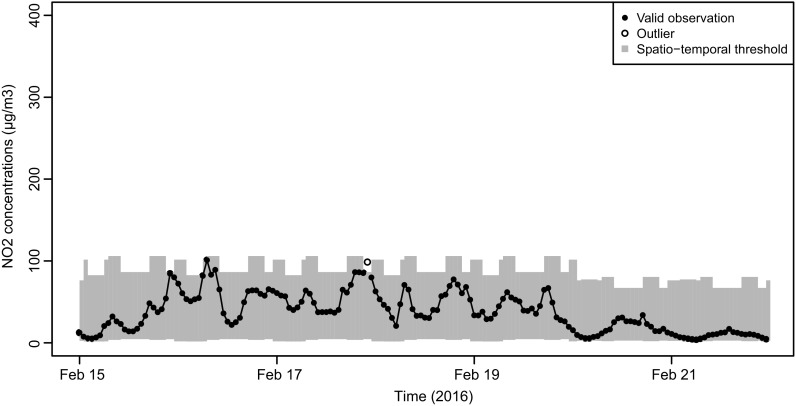
NO_2_ concentrations measured by a conventional monitor at an urban traffic location. Filled circles indicate non-outlying observations; unfilled circles indicate outliers using *z* = 2.97. The gray bars indicate the threshold values for each temporal class, for urban traffic conventional monitors

Similar results were found using hourly NO_2_ observations of 2016 from the two conventional monitors. The total number of outliers detected was 0.3% of the dataset, which varied from 0 to 0.7% depending on the temporal class. In Fig. 7, we observe a different pattern in the spatio-temporal thresholds compared to the threshold pattern of the airboxes (Figs. 5 and 6). Note that for the conventional monitors, we also observe positive lower threshold values, though close to zero. In Fig. 7, we identify one outlier, which occurred in the off-peak hour period after the evening rush hour. This period after the evening rush hour is the period in which most outliers occurred for the conventional monitors.

**Fig. 8:**
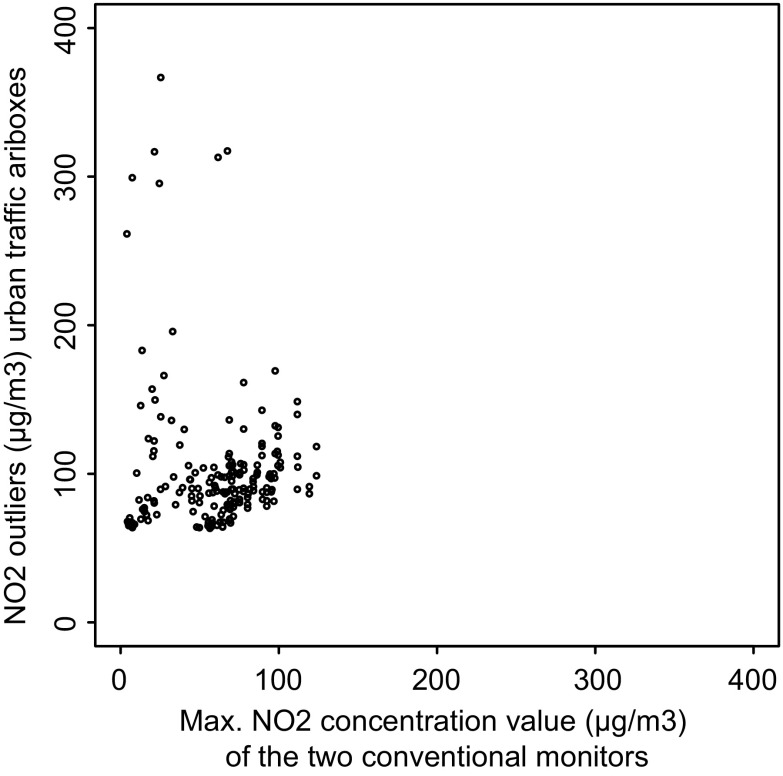
Scatterplot of traffic airbox outliers vs. the maximum NO_2_ concentration measured at the same moment in time by the two conventional monitors located in traffic sites

We compared the outliers in the traffic airboxes with the NO_2_ concentrations measured with the conventional monitors at the same time. A scatterplot is shown in Fig. 8. The plot shows many observations down-right in the plot that have similarly high concentrations measured by the airbox and the conventional monitor, though at different locations. Some outliers occurred in multiple airboxes at the same time. This may be an indication of a pollution event that has an effect on the entire city. Down-left in the plot, we find observations that are considered outliers by the airboxes, but are within normal range of concentrations according to the conventional monitors. These could be errors or very local air pollution events. In the upper part of the plot, we find very high concentrations measured by the airbox which are higher than any value measured by the conventional monitor in the entire year. These are most likely errors.

## Discussion

The results show that the spatio-temporal classification of NO_2_ concentration values in an urban sensor network is a simple outlier detection method in an area with high spatial and temporal variability of air pollutant concentrations. The number of outliers detected using the classification (0.1–0.5% for the airboxes and 0–0.7% for the conventional monitors) matches expectation when using *z* = 2.97 as a threshold for the number of standard deviations, including 99.7% of the observations under the assumption of a normal distribution. The value of *z* can be tuned depending on the application. A lower value of *z* will result in more concentration values to be considered outliers. Brown and Brown (2012) suggest that the choice of the threshold value should be a trade-off between the extra work associated with investigating false positives, i.e., observations falsely detected as outliers, and the likelihood of false negatives, i.e., true outliers that are not detected.

We aimed to compare the above procedure with kriging-based outlier detection (Zhang et al. 2012). We found that the NO_2_ concentrations vary over shorter distances than the distances between measurement locations, resulting in a pure noise variogram. Sampling NO_2_ over shorter distances, e.g., within a few meters, might make it possible to apply kriging-based outlier detection methods, especially when including covariates such as road distance and wind direction into the model.

Air pollutant concentrations are generally considered lognormally distributed (Ott 1990). Applying the proposed outlier detection method on log-transformed NO_2_ concentrations would however result in an implausible number of outliers detected on the left side on the distribution (99.5%) compared to the right side of the distribution (0.5%). Instead, we are mostly interested in high peaks in the data, which can be used to detect air pollution events and errors. Therefore, we used a square root transformation of the NO_2_ concentration data.

The temporal classification used in this analysis is mostly based on expected traffic during certain hours of the day. Other factors that may influence the temporal variability in NO_2_ concentrations are meteorological factors such as wind speed, wind direction, air pressure, temperature, and solar radiation. An analysis of seasonal and diurnal variation at a UK city is presented by Bigi and Harrison (2010). NO_2_ concentrations in Europe tend to be higher in the winter than in the summer season. Hence, observations in the summer season had a lower chance to be detected as outliers by our method. Our method can be expanded by defining more classes, for example, taking into account season and meteorological factors, or by taking into account temporal autocorrelation. For simplicity reasons, we used full year data for the current paper.

Public holidays occurring on a weekday are classified as weekdays, although the concentrations are likely lower, and therefore more similar to weekend concentrations. A visual analysis of the data showed that there was no increase in low-peak outliers during such holidays. High-peak outliers occurred and were also detected during the weekday holidays.

In this study, we aggregated the NO_2_ concentrations to hourly values. Using 10-min data, the outlier detection method would give more detailed instances of outliers compared to using hourly data. The results of 10-min outlier detection should be interpreted differently from the results of hourly outlier detection. In hourly outlier detection, peaks occurring as a result of a strongly emitting vehicle passing by are more likely to be averaged out as they may occur every hour. In 10-min data, such peaks are more likely to be considered outliers. Hourly outliers give a better overview of hours in which there is an abnormal number of peaks rather than showing individual peaks, as in the case of 10-min outlier detection.

**Fig. 9:**
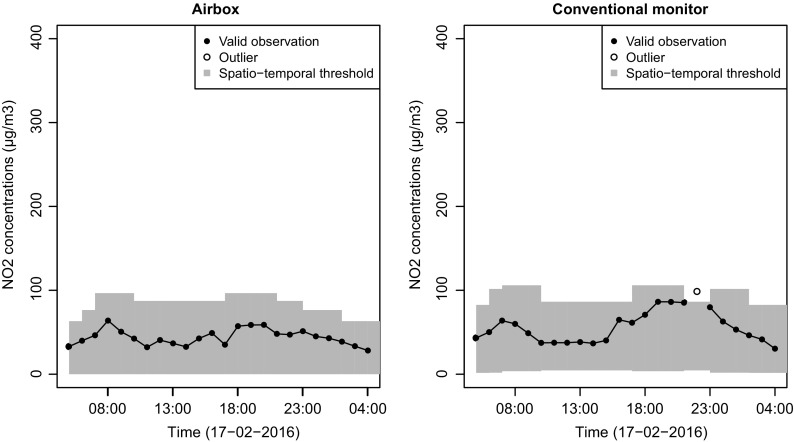
Comparison of NO_2_ concentrations measured by an airbox (left) and a conventional monitor (right) on a weekday at urban traffic locations. Filled circles indicate non-outlying observations; unfilled circles indicate outliers using *z* = 2.97. The gray bars indicate threshold values for each temporal class and are specific for each dataset, characterized by a spatial class and measurement instrument

For the conventional monitors, the largest number of outliers was found during the off-peak period after the evening rush hours. Comparing the daily threshold pattern of the airbox to that of the conventional monitor on a weekday (Fig. 9), both at an urban traffic location, we see that the upper threshold of the airbox in off-peak periods (87.3 μg m^−3^) lays between the upper threshold of rush hours (96.6 μg m^−3^) and the upper threshold of transition periods (76.5 μg m^−3^). For the conventional monitor, the upper threshold for off-peak periods (86.4 μg m^−3^) is below the threshold for both rush hours (106 μg m^−3^) and transition periods (101.6 μg m^−3^). The threshold for off-peak periods is calculated using the observations between morning rush hour and evening rush hour (9:01–16:00 UTC time) combined with the observations after evening rush hour (20:01–22:00 UTC time). For the airboxes, this is alright because the concentrations are within a similar range. The authorative monitors, however, still measure high concentrations for 2 h after the evening rush hour. This leads to underestimation of the threshold after evening rush hour. The cause of this difference is unclear, but most likely it is caused by differences between the sensor system of the airbox and the conventional monitor, and could be solved by defining different temporal classes depending upon the measurement instrument used.

The spatial classification method has been applied to the city of Eindhoven, the Netherlands. The spatio-temporal variability of NO_2_ concentrations in this city is determined mainly by road traffic, like in many European cities (Cyrys 2012). The spatial classification used in this analysis, distinguishing between urban background locations and urban traffic locations, is based upon this spatial variability. In Asian cities where, for example, industry plays a major role in the spatio-temporal variability of NO_2_ concentrations (Cui 2016), other classifications may be more relevant.

The proposed method for outlier detection using a spatio-temporal classification of the NO_2_ variability was found useful for distinguishing outliers in an area with high spatial and temporal variability of air pollutant concentrations. This provides a basis for future work on distinguishing between types of outliers, e.g., errors and events. Air pollution events are often characterized by lasting for a period of time, which would lead to a number of outliers in a row for the same sensor. Such events can also be characterized by covering a large area in space. The occurrence of outliers at multiple locations at the same moment may indicate such an event.

The method provides a useful outlier detection method for those involved in urban air quality sensor networks. Its use in other fields of environmental variables with a high spatial and temporal variability is to be further investigated and will largely depend on the ability to classify the observations in various spatial and temporal categories.

Future research is needed in order to deal with the application of this method for (near) real-time outlier detection, in which each new observation can be compared to previous observations in the same spatio-temporal class. By using a moving average over the last hour, applied every 10 min, the method can be applied to (near) real-time data. Its applicability is currently mostly limited by the computation time, which is too long for real-time outlier detection. This may in the future be improved by using higher computation power or smaller datasets, or a combination of these two.

## Conclusions

We presented a novel method for outlier detection in urban air quality sensor networks, based on dividing the observations in two spatial and eight temporal classes. Each of the 16 resulting spatio-temporal classes represents a range of typical air pollutant concentrations for this class. By finding outliers in each class separately, the spatio-temporal variability in concentrations is maintained. In doing so, this work addressed an important challenge in outlier detection in urban areas.

In our analysis using hourly NO_2_ data from an air quality sensor network in Eindhoven, the Netherlands, we detected 0.1–0.5% of outliers using a 99.7% confidence interval. The size of the confidence interval can be changed depending on the application. The non-normality of air pollutant concentrations is taken into account by using a truncated normal distribution of square-root-transformed concentrations. The method is easy to implement and simple to adjust to other cities and pollutants by choosing spatio-temporal classes based on the sources of the air pollutants.

This research is a first step in outlier detection of NO_2_ concentrations in urban areas. The detected outliers are unusually high concentrations, which can be either errors or events. Expert knowledge is however required to evaluate each outlier and decide on its treatment. Further research is needed with a focus on automatically distinguishing errors from events and (near) real-time outlier detection.

## Electronic Supplementary Material

ESM 1 (PDF 257 kb)
