# Supplementary material for: Outlier Detection in Urban Air Quality Sensor Networks
Source: Water Air Soil Pollut. 2018 Mar 8;229(4):111. doi: 10.1007/s11270-018-3756-7 (PMC5843703; doi:10.1007/s11270-018-3756-7)
Supplement: Supplementary file 1 — (PDF 257 kb) [file 11270_2018_3756_MOESM1_ESM.pdf]

Supplementary material for “Outlier detection in urban air quality sensor networks”

V.M. van Zoest [a, *], A. Stein [a], G. Hoek [b]

[a] Faculty of Geo-Information Science and Earth Observation (ITC), University of Twente, PO Box 217, 7514 AE, Enschede, The Netherlands

[b] Institute for Risk Assessment Sciences (IRAS), Utrecht University, PO Box 80178, 3508 TD, Utrecht, The Netherlands

* Corresponding author. Email address: v.m.vanzoest@utwente.nl.

S1. Supplementary table 1

Mean n_K (+/- standard deviation t_K) of the distribution underlying the truncated normal distribution of each spatio-temporal class. Note that these are not actual concentration values, but transformed concentration values. Threshold values are obtained by $n_K \pm z \times t_K$ and back-transformation of the resulting values using equation (9).

	**Urban traffic**		**Urban background**	
	Week	Weekend	Week	Weekend
**Rush hours**	5.26 (+/- 1.55)	4.54 (+/- 1.47)	4.67 (+/- 1.48)	4.05 (+/- 1.31)
**Off-peak hours**	5.02 (+/- 1.47)	4.50 (+/- 1.45)	4.46 (+/- 1.39)	4.01 (+/- 1.31)
**Night hours**	4.30 (+/- 1.25)	4.22 (+/- 1.28)	3.97 (+/- 1.27)	3.83 (+/- 1.28)
**Transition hours**	4.76 (+/- 1.36)	4.39 (+/- 1.30)	4.33 (+/- 1.34)	3.99 (+/- 1.21)
